# ﻿*Miradessus* gen. nov. from South America described for two species previously in *Amarodytes* Régimbart, 1900 and two new species (Arthropoda, Insecta, Coleoptera, Adephaga, Dytiscidae, Hydroporinae, Bidessini)

**DOI:** 10.3897/zookeys.1176.104980

**Published:** 2023-08-22

**Authors:** Kelly B. Miller, Cesar J. Benetti, Mariano C. Michat

**Affiliations:** 1 Department of Biology and Museum of Southwestern Biology, University of New Mexico, Albuquerque, NM 87131-0001, USA University of New Mexico Albuquerque United States of America; 2 Departamento de Biodiversidad y Gestión Ambiental, Facultad de Ciencias Biológicas y Ambientales, Universidad de León, Campus de Vegazana, 24071, León, Spain Universidad de León León Spain; 3 Coordenação de Biodiversidade, Programa de Pós-Graduação em Entomologia (PPGEnt), Instituto Nacional de Pesquisas da Amazônia (INPA), Av. André Araújo 2936, CEP 69067-375, Manaus, AM, Brazil Instituto Nacional de Pesquisas da Amazônia (INPA) Manaus Brazil; 4 Faculty of Exact and Natural Sciences, Department of Biodiversity and Experimental Biology, Laboratory of Entomology, Institute of Biodiversity and Experimental and Applied Biology (IBBEA), CONICET-University of Buenos Aires, Buenos Aires, Argentina CONICET-University of Buenos Aires Buenos Aires Argentina

**Keywords:** Diving beetle, South America, taxonomy, water beetle

## Abstract

*Miradessus***gen. nov.** is described for two previously described species, *Amarodytespulchellus* Guignot, 1955 from Colombia, with new records from Venezuela, and *A.plaumanni* Gschwendtner, 1935, from Brazil, and two previously unknown species, *Miradessusbeni***sp. nov.**, from Bolivia and Peru, and *Miradessusrikae***sp. nov.** from Ecuador. The genus is characterized by 1) occipital line absent; 2) basal pronotal striae present; 3) basal elytral stria absent; 4) sutural elytral stria absent; 5) transverse carina on elytral epipleuron at humeral angle absent; 6) distinct marginal bead on anterior clypeal margin absent; and 7) male median lobe deeply multilobed with a dorsal portion separate from a unilobed or bilobed ventral portion.

## Abstract (es)

Se describe a *Miradessus***gen. nov.** para dos especies previamente descriptas, *Amarodytespulchellus* Guignot, 1955 de Colombia, con nuevos registros de Venezuela, y *A.plaumanni* Gschwendtner, 1935, de Brasil, y dos especies previamente desconocidas, *Miradessusbeni*, **sp. nov.**, de Bolivia y Perú, y *Miradessusrikae*, **sp. nov.**, de Ecuador. El género se caracteriza por 1) línea occipital ausente; 2) estría pronotal basal presente; 3) estría elitral basal ausente; 4) estría elitral sutural ausente; 5) carena transversa en el ángulo humeral del epipleuron elitral ausente; 6) reborde marcado en el margen anterior del clípeo ausente; y 7) lóbulo medio del macho profundamente multilobado con una porción dorsal separada de una porción ventral unilobada o bilobada.

## ﻿Introduction

The tribe Bidessini Sharp, 1880 includes an unusually large proportion of genera and species of Dytiscidae (Miller and Bergsten 2016). They occur in a great many habitats throughout the world (Miller and Bergsten 2016). New genera have been regularly discovered in recent years through field expeditions in new regions or habitats (e.g. hygropetric or subterranean) and reexamination of historically recognized genera (Miller and Spangler 2008; Hendrich and Balke 2009; Hendrich et al. 2009; Miller and García 2011; Miller 2012, 2016a; Miller and Short 2015; Miller and Wheeler 2015; Balke et al. 2017).

During a revisionary investigation of the genus *Amarodytes* Régimbart, 1900 by the authors it became clear that there are several groups of Bidessini species historically involved in the genus that are seemingly more closely related to other groups in Bidessini than to each other. The type species, *A.percosioides* Régimbart, 1900, is part of a group that includes species with single-segmented lateral lobes that are related to *Hydrodessus* J. Balfour-Browne, 1953 and *Peschetius* Guignot, 1942, which also have single-segmented lateral lobes (Miller et al. 2006; Miller and Bergsten 2014, 2016, 2023; Miller 2016b). However, other species assigned to *Amarodytes* were found to have two-segmented lateral lobes, and, therefore, are misplaced in the genus (Benetti and Régil Cueto 2004). Two of these species were described as *Amarodytespulchellus* Guignot, 1955 and *Amarodytesplaumanni* Gschwendtner, 1935. Other specimens recently examined from Bolivia and Peru are similar to *A.pulchellus*, but represent a different, undescribed species described here. Finally, two specimens from Ecuador were also found to represent an unknown species. These species together possess a unique set of character states and cannot be placed into any existing Bidessini genus, nor do they belong in *Amarodytes*. For this reason, a new genus is here erected to include them. The systematics of *Amarodytes* will require additional attention to address the *A.percosioides*-, *A.duponti*-, and *A.segrix*-groups which seem unlikely to be appropriately placed in the same genus (Benetti and Régil Cueto 2004).

## ﻿Materials and methods

Methods for dissections and other treatment of specimens largely follow recommendations by Miller and Bergsten (2016).

### ﻿Materials

Specimens from nearly every genus of Bidessini were examined, including multiple species from many of them. Specimens of relevant species treated herein were examined primarily from the following collections:

**MIZA**Museo del Instituto de Zoología Agrícola Francisco Fernández Yépez, Universidad Central de Venezuela, Maracay, Venezuela (L. Joly);

**MSBA**Museum of Southwestern Biology, Division of Arthropods, University of New Mexico, Albuquerque, NM, USA (K.B. Miller);

**OLML**Oberösterreichisches Landesmuseum, Linz, Austria (M. Schwarz);

**SEMC**Snow Entomological Collection, University of Kansas, Lawrence, KS, USA (A.E.Z. Short);

**USNM**United States National Museum, Department of Entomology, Washington, DC, USA (S. Brady);

**ZSMG**Zoologische Staatssammlung, Munich, Germany (M. Balke).

### ﻿Measurements

Measurements were taken with an ocular scale on a Zeiss Discovery V8 dissecting microscope at 50× magnification. Attempts were made to measure the most variable specimens in size and shape to determine the extent of that variation. Measurements include: 1) total length (TL), 2) greatest width across elytra (GW), 3) greatest pronotal width (PW), 4) greatest width of the head (HW), 5) distance between the eyes (EW), 6) greatest length of metatrochanter (RL), and 7) greatest length of metafemur (FL). The ratios TL/GW, HW/EW, and FL/RL were calculated to provide an indication of relative size and shape of certain structures.

### ﻿Photos and illustrations

Methods for images largely follow Miller and Bergsten (2016).

## ﻿Results

### ﻿ Miradessus

Taxon classificationAnimaliaColeopteraDytiscidae

Miller, Benetti & Michat gen. nov.

D4291A62-A4F8-5142-9C14-9590B6809481

https://zoobank.org/1BF90E3E-44AC-4D82-A84C-8953ADCF7798

Figs 1–8 , 9–17 , 18–29 , 30

#### Type species.

*Amarodytespulchellus* Guignot, 1955 by current designation.

#### Diagnosis.

**Figures 1–8.**
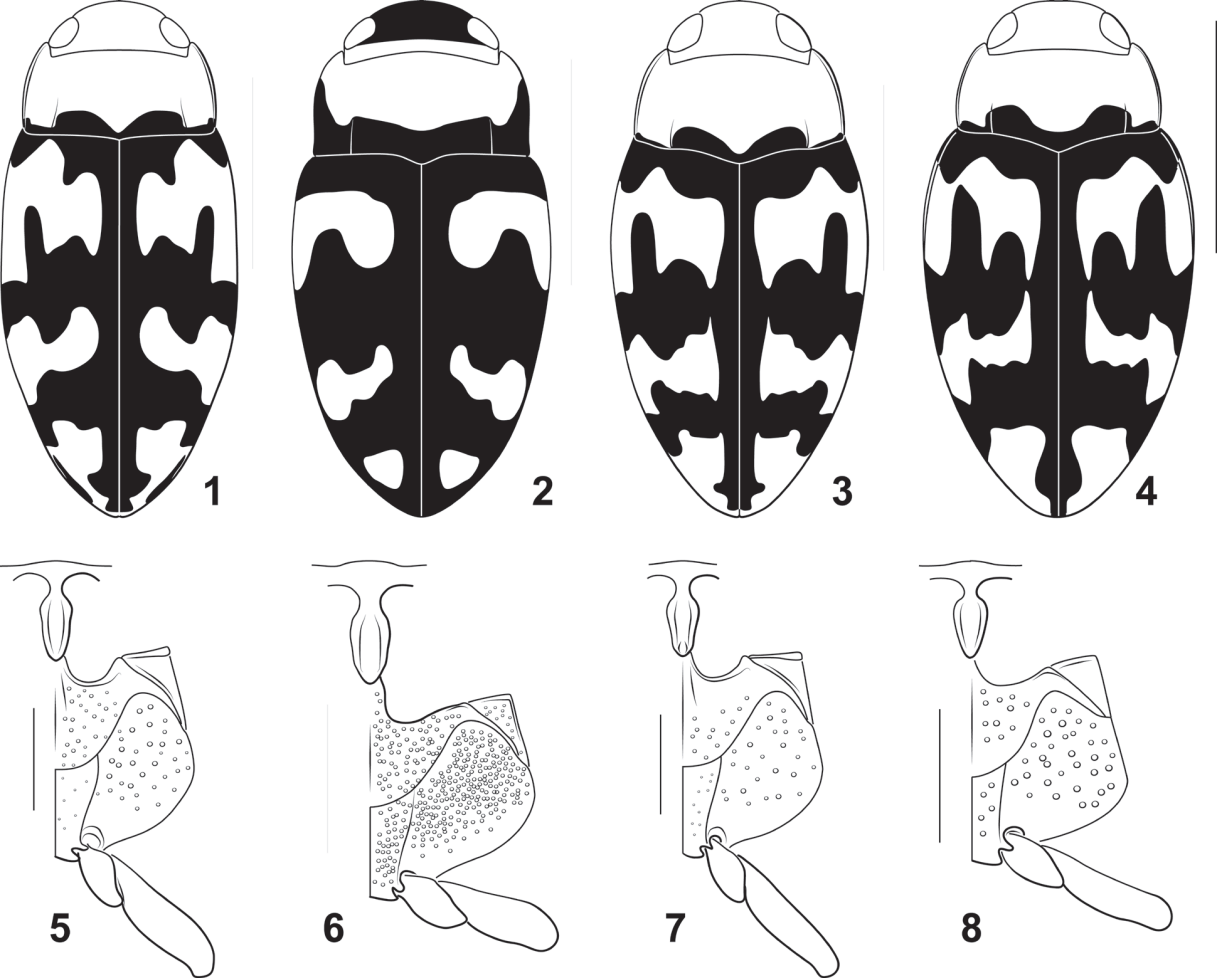
*Miradessus* species **1–4** dorsal habitus **1***M.beni***2***M.plaumanni***3***M.pulchellus***4***M.rikae***5–8** prosternal process left surfaces of metaventrite metacoxa metatrochanter and metafemur **5***Miradessusbeni***6***M.plaumanni***7***M.pulchellus***8***M.rikae*. Scale bars: 1.0 mm (**1–4**); 0.5 mm (**5–8**).

**Figures 9–17.**
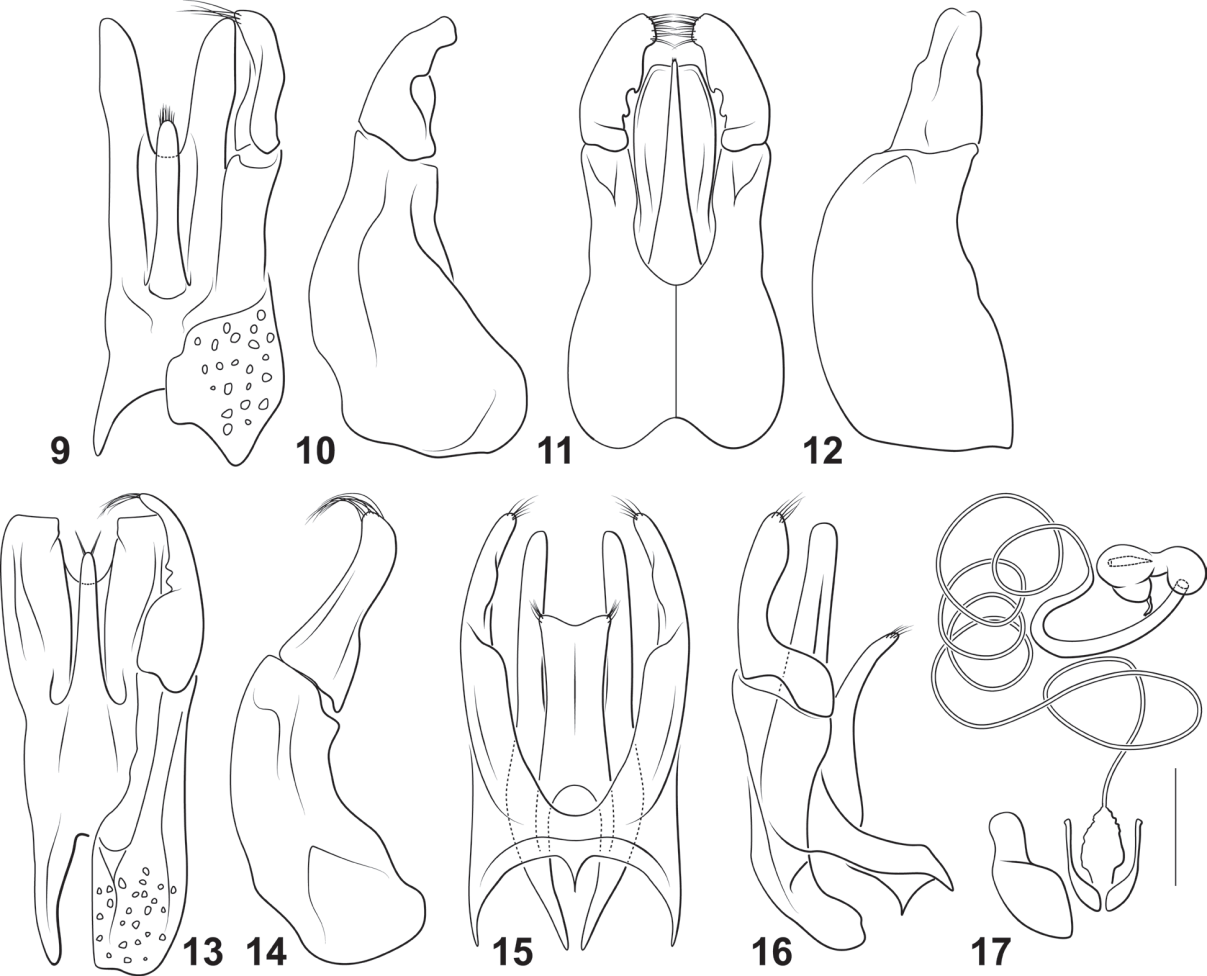
*Miradessus* species **9, 10***M.beni*, male aedeagus **9** median lobe and left lateral lobe, dorsal aspect **10** right lateral lobe, right lateral aspect **11, 12***M.plaumanni*, male aedeagus **11** median lobe and lateral lobes, dorsal aspect **12** right lateral lobe, right lateral aspect **13, 14***M.pulchellus*, male aedeagus **13** median lobe and left lateral lobe, dorsal aspect **14** right lateral lobe, right lateral aspect **15, 16***M.rikae*, male aedeagus **15** median lobe and lateral lobes, dorsal aspect **16** median lobe and right lateral lobe, right lateral aspect **17***M.pulchellus*, female genitalia, ventral aspect. Scale bar: 0.25 mm.

*Miradessus* belongs to the tribe Bidessini based on the presence of bisegmented lateral lobes (Figs 10, 12, 14, 16) and a spermathecal spine (Fig. 17). Within Bidessini, the genus differs from most other genera in the following character combination: 1) occipital line absent (Figs 1–4), 2) basal pronotal striae present (Figs 1–4), 3) basal elytral stria absent (Figs 1–4), 4) sutural elytral stria absent (Figs 1–4), 5) transverse carina on elytral epipleuron at humeral angle absent, and 6) distinct marginal bead on anterior clypeal margin absent (Figs 1–4). Other genera share these features including *Novadessus* Miller, 2016, *Bidessodes* Régimbart, 1900, *Neobidessodes* Hendrich & Balke, 2009, the *Amarodytesduponti* group, and *Amarodytessegrix* Guignot, 1950. But *Miradessus* differs from all of these in the distinctive shape of the male genitalia (Figs 9–16). The male median lobe is very characteristically apically multilobed with a single, dorsal elongate and slender portion and a ventral section that is broad and bilobed (Figs 9, 11, 15) or flattened and elongate (Fig. 13). The lateral lobes are robust, and the bases are large and, in at least some species, covered with conspicuous tubercles (Figs 9, 13). Members of the genus are also superficially quite distinctive from other Bidessini (see more below under Discussion).

#### Etymology.

This genus is named *Miradessus* from the Latin *miror*, meaning to admire, for the impressive color pattern of these beetles, and *dessus*, a common root for genera in the tribe Bidessini (gender masculine).

#### Discussion.

The species in this new genus are characterized by lateral lobes that are distinctly two-segmented like the majority of Bidessini taxa (Biström 1988; Miller et al. 2006; Miller and Bergsten 2014; 2016; Miller 2016b;). Members of typical *Amarodytes* (including the type species, *Amarodytespercosioides* Régimbart, 1900) have single-segmented lateral lobes and belong within a clade sister to a clade characterized by two- or three-segmented lateral lobes (Benetti & Miller unpublished). Within the bisegmented lateral-lobe clade, these specimens do not fit well into any other genera (see Diagnosis above). They share some character combinations with *Novadessus*, *Bidessodes*, *Neobidessodes*, the *Amarodytesduponti* group, and *Amarodytessegrix*. In some ways they are most superficially similar to members of *Bidessodes* Régimbart, but specimens in that genus have series of very fine denticles along the posterior margins of the abdominal ventrites (Miller 2017), which are lacking in *Miradessus*, and also have distinctly different male genitalia (Miller 2017). *Miradessus* are similar to Neobidessodes, but that genus has simple male median lobes unlike the multilobed condition in *Miradessus* (Figs 9, 11, 13, 15). Otherwise, they are not similar to other genera in the tribe and are unique because of the prominently apically multilobed male median lobe (Figs 9, 11, 13, 15) which is not found in other genera of Bidessini.

### ﻿ Miradessus beni

Taxon classificationAnimaliaColeopteraDytiscidae

Miller, Benetti & Michat sp. nov.

34C12EF8-D228-5E09-82A3-EA2FCBDCF4D0

https://zoobank.org/6A3EEA04-9DEA-41DA-A2F4-40E795270841

Figs 1 , 5 , 9 , 10 , 18–20 , 30

#### Type locality.

Bolivia, Departmento de La Paz, Provincia de Abel Iturralde, San Miguel del Bala, 14°30.602'S, 67°29.555'W.

#### Diagnosis.

This species and *M.pulchellus* are extremely similar externally, with similar overall shape, color pattern, and other features (Figs 1, 3). The external differences between them are subtle, including the shape of the prosternal process, which is more prominently laterally carinate and deeply sulcate in *M.pulchellus* than in *M.beni*. Also, the ventral surface is darker in most specimens of *M.pulchellus* than in *M.beni*. The main differences between these species are in the male genitalia, which are distinctive and characteristic. Both have the median lobe trilobed (with two side portions and a middle portion), but in *M.pulchellus* the middle portion is nearly as long as the side portions and the apex has a distinct, fine pencil of setae on each side that are divergent (Fig. 13). In *M.beni* the middle portion is considerably shorter than the side portions and has a series of setae along its apex (Fig. 9). In lateral aspect, the lateral lobe of *M.pulchellus* has the apical segment nearly as long as the basal segment and it is curved ventrad (Fig. 14). In *M.beni* the lateral lobe has the apical segment only about 1/3 the length of the basal segment and it is curved dorsad (Fig. 10).

#### Description.

***Measurements*.**TL = 2.7–2.8 mm, GW = 1.3–1.4 mm, PW = 1.0–1.1 mm, HW = 0.7–0.8 mm, EW = 0.3–0.4 mm, TL/GW = 2.0–2.1, HW/EW = 1.8–1.9, FL/RL = 2.2–2.3.

***Habitus*.** Body shape elongate-oval, lateral outline somewhat discontinuous between pronotum and elytron, posterior apex narrowly rounded (Fig. 1).

***Coloration*** (Fig. 1). Head yellow-orange. Pronotum yellow-orange with a narrow, rounded lobe of black broadly along each side of posterior margin. Elytron maculate, yellow-orange and black, margins of maculae strongly demarcated; with large, transverse yellow-orange regions anteriorly, medially, and apically, not extending medially to suture, with narrow band of black along entire length of suture, margins of maculae irregular and variously lobed (Fig. 1). Ventral surfaces mostly orange; legs, epipleuron, and lateral areas of prothorax and head lighter orange-yellow, some sutural margins darker, infuscate, mesothoracic ventrites and prosternal process strongly infuscate to nearly black.

***Sculpture and structure*.** Head shiny and smooth, nearly impunctate, with small micropunctures sparsely distributed; eyes moderately large (HW/EW = 1.8–1.9); antennae slender, unmodified. Pronotum with lateral margins moderately curved anteriorly, shallowly curved posteriorly; with narrow bead along entire margin; surface shiny, moderately and evenly punctate; lateral pronotal plica weakly impressed, sublinear, extending about 1/3 distance across pronotum. Elytron with lateral margin evenly and broadly rounded; surface shiny, moderately and evenly punctate. Prosternum medially moderately broad, medially not protruberant, mediolaterally somewhat granular; prosternal process moderately broad medially with slight tubercle, apical blade large, covered with fine setae, laterally with prominent rounded ridges along entire length, medially longitudinally sulcate, lateral margins slightly convergent to narrowly rounded apex (Fig. 5). Metaventer and metaventral wings smooth and shiny, covered with shallow punctures (Fig. 5). Metacoxa with medial portion moderately broad, metacoxal lines distinct, evenly divergent anteriorly to posterior margin of metaventrite, lateral portion large, medially and anteriorly covered with shallow punctures; metatrochanter about 1/3 length of metafemur (Fig. 5). Abdominal ventrites impunctate except II and III laterally with shallow, indistinct punctures; VI strongly concave in lateral aspect, apex pointed.

***Male genitalia*.** Median lobe in ventral aspect broad, trilobed, with dorso-medial, elongate, slender, apically pointed ramus and another ventral, elongate, apically bilobedramus, with each ramus elongate and slender, medial dorsal ramus much shorter than ventral ramus (Fig. 9); lateral lobe in lateral aspect robust, basal segment very broad, robust, apical segment short, robust, curved dorsad, apex obliquely truncate (Fig. 10).

***Sexual dimorphism*.** Males have the pro- and mesotarsomeres I–III slightly but distinctly broader than in females. Abdominal ventrite VI distinctly concave in lateral aspect in females, but medially more expanded and apically somewhat depressed in males.

***Variation*.** There is some minor variation in shape and extent of coloration of the dorsal and ventral surfaces but otherwise specimens are similar.

#### Etymology.

This species is named *beni* after the name Río Beni, the river at the type locality of the species. The name is a noun in apposition.

#### Distribution.

*Miradessusbeni* is known from the type locality in lowland Departmento de La Paz, Bolivia and from two sites in lowland Peru (Fig. 30).

#### Habitat.

The type locality is a heavily forested area of the Andean foothills next to a large river. However, nothing is known of the specific collection habitat of this species.

#### Material examined.

**Figures 18–29.**
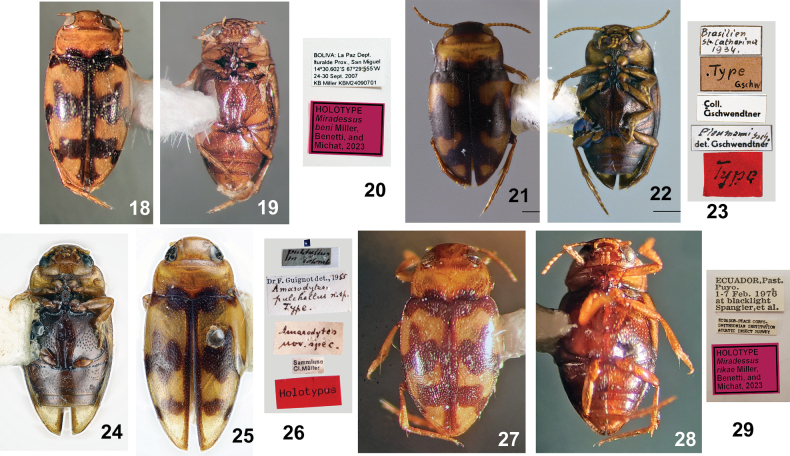
*Miradessus* species, primary type specimens and labels **18–20***M.beni*, holotype specimen **18** dorsal habitus **19** ventral habitus **20** specimen labels **21–23***M.plaumanni*, lectotype specimen **21** dorsal habitus **22** ventral habitus **23** specimen labels **24–26***M.pulchellus*, holotype specimen **24** dorsal habitus **25** ventral habitus **26** specimen labels **27–29***M.rikae*, holotype specimen **27** dorsal habitus **28** ventral habitus **29** specimen labels. **21–23** courtesy of M. Schwarz (OLML) **24–26** courtesy of M.A. Balke (ZSMG).

***Type material*. *Holotype*** (Figs 18–20) in MIZA, male labeled, “BOLIVIA: La Paz Dept. Ituralde [sic] Prov., San Miguel 14°30.602'S, 67°29.555'W, 24–30 Sept. 2007 KB Miller KBMC24090701/ HOLOTYPE *Miradessusbeni* Miller, Benetti & Michat, 2023 [red label with black line border].”

***Paratypes***, 41 total; 6 paratypes (MIZA, MSBA) labeled same as holotype;” 8 paratypes (USNM) labeled, “PERU:Dpt. Cuzco Prov. Quispicanchis Quincemil 6-II-X-1976 Robert Gordon”; 12 paratypes (ZSMG) labeled, “Bidessus cruciatus ? [handwritten] / Amarodytespulchellus [handwritten] / Peru, Prov. Huanuco, Rio Yuyapichis, Biol. Stat. Panguana. Östl. Ort. 9°37'S, 74°56'W 6.–17.April 2003, Malaise [handwritten], leg. H., J. u. E. -G. Burmeister”; 2 paratypes (ZSMG) labeled, “PERU, Dept. Huanuco, Panguana ACP, Rio Yuyapichis 9°37'S – 74°56'W, at blacklight, XII.2015 J. Monzon leg.”;4 paratypes (ZSMG) same as previous except “…/230 m, blacklight trap/ Hydrodessus sp. 1 [handwritten], Hendrich dt. 2020”; 3 paratypes (ZSMG) same as previous except “…/I.2016”; 4 paratypes (ZSMG) same as previous except “…/V–VI.2017/ Dytiscidae ?Hydrodessus sp. [handwritten], H.J. Bremer det. 2018”; 2 paratypes (ZSMG) same as previous except “…/Lux – 20.9.–9.10.2007 leg. Burmeister.” Each paratype with “…/PARATYPE *Miradessusbeni* Miller, Benetti & Michat, 2023 [blue label with black line border].”

### ﻿ Miradessus plaumanni

Taxon classificationAnimaliaColeopteraDytiscidae

(Gschwendtner, 1935) comb. nov.

304052B9-06A2-5BCA-A51D-B29078A1E906

Figs 2 , 6 , 11 , 12 , 21–23 , 30

Amarodytes plaumanni Gschwendtner, 1935: 152; Young 1969: 2; Trémouilles 1995: 47; Nilsson and Hájek 2022: 101.
Bidessus plaumanni : Blackwelder 1944: 76.

#### Type locality.

Brazil, Santa Catarina State, Nova Teutônia.

#### Diagnosis.

This species differs considerably from the other known species in the genus. The anterior area of the dorsal surface of the head is testaceous in this species (Fig. 2), but pale yellow in the other species (Figs 1, 3, 4). The ventral portion of the male median lobe in *M.plaumanni* is broad and unilobate (Fig. 11) instead of strongly bilobate as in the other species (Figs 9, 13, 15). Other differences from other species in the group include: 1) lateral pronotal margins nearly straight posteriorly (Fig. 2) instead of broadly curved (Figs 1, 3, 4), 2) the pronotum and elytron different in color pattern (Fig. 2) from the other, more uniformly-patterned species (Figs 1, 3, 4), and 3) the lateral portions of the metaventrite and metacoxa more coarsely and densely punctate (Fig. 6) than in other species (Figs 5, 7, 8). The general shape and dorsal coloration of specimens are rather different as well (Figs 1–4).

#### Description.

***Measurements*.**TL = 2.3 mm, GW = 1.2 mm, PW = 0.9 mm, HW = 0.7 mm, EW = 0.4 mm; TL/GW = 2.00, HW/EW = 1.7, FL/RL = 1.9.

***Habitus*.** Body shape elongate-oval, lateral outline discontinuous between pronotum and elytron, posterior apex somewhat acuminate (Fig. 2).

***Coloration*** (Fig. 2). Head brown with a narrow orange band along posterior margin. Pronotum yellow-orange with broad dark band along posterior margin and narrow band along lateral margins. Elytron maculate, yellow-orange and black, margins of maculae strongly demarcated; with transverse yellow-orange regions anteriorly, medially and apically, not extending to suture, with broad band of black along entire length of suture, margins of basal maculae posteriorly bilobed. Ventral surfaces mostly dark orange-brown; legs, epipleuron, and ventral areas of prothorax and head lighter orange-yellow, some sutural margins darker, infuscate, mesothoracic ventrites and prosternal process strongly infuscate.

***Sculpture and structure*.** Head smooth, nearly impunctate, with small micropunctures sparsely distributed; eyes large (HW/EW = 1.7); antennae slender, unmodified. Pronotum with lateral margins moderately curved anteriorly, almost straight posteriorly; with narrow bead along entire margin; surface moderately punctate, punctures more concentrate along posterior margin; lateral pronotal plica strongly impressed, almost straight, extending more than 1/3 distance across pronotum. Elytron with lateral margin evenly and broadly rounded; surface finely and evenly punctate, with a slightly marked line of punctures with short setae extending medially from base to apex. Prosternum medially moderately broad, medially not protruberant, mediolateral surface somewhat granular; prosternal process moderately broad medially with slight tubercle, apical blade large, laterally with low rounded ridges along entire length, medially longitudinally shallowly sulcate, lateral margins almost straight to narrowly rounded apex (Fig. 6). Metaventer and metaventral wings smooth and shiny, covered with coarse, dense, and evenly impressed punctures. Metacoxa with medial portion moderately broad, metacoxal lines distinct, evenly divergent anteriorly to posterior margin of metaventer; lateral portion large, evenly covered with dense, coarse punctures; metatrochanter about 1/3 length of metafemur (Fig. 6). Abdominal ventrites finely punctured with fine setae.

***Male genitalia*.** Median lobe in ventral aspect broad, with medial, dorsal elongate very slender, apically sharply pointed portion and ventral, broad, flattened, apically broadly rounded portion, dorsal portion slightly longer than ventral portion (Fig. 11); lateral lobe in lateral aspect robust, basal segment very broad, robust, apical segment short, robust, straight, apex broad, slightly bilobed (Fig. 12).

***Sexual dimorphism and variation*.** Males have the pro- and mesotarsomeres I–III slightly but distinctly broader than in females.

#### Distribution.

**Figure 30.**
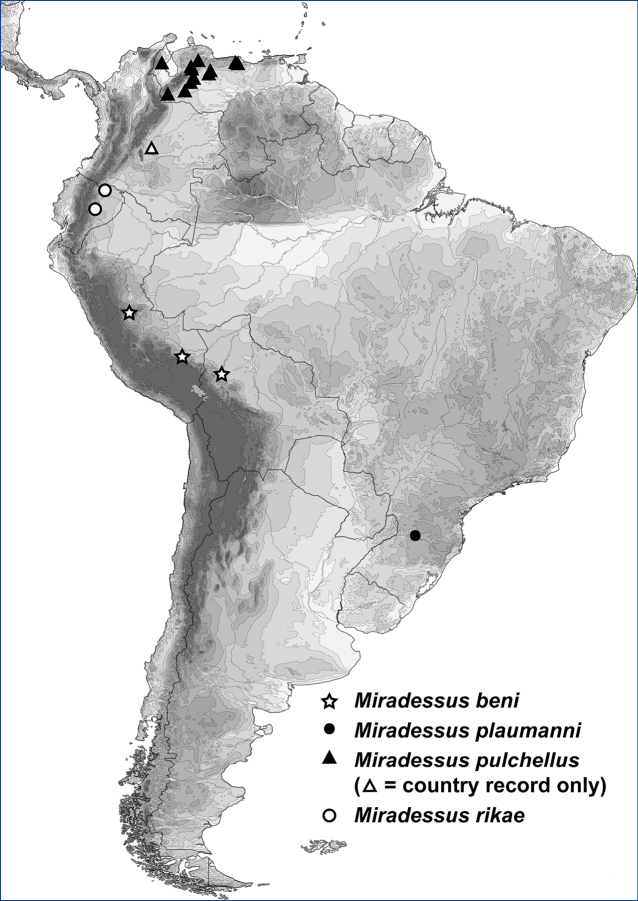
*Miradessus* species, distributions.

This species is only known from the type locality, Brazil, Santa Catarina state, Nova Teutônia (Fig. 30).

#### Habitat.

Nothing is known of the habitat of this species.

#### Material examined.

***Type specimens*.** Five syntype specimens are in OLML. One male specimen is labeled, “Brasilien S. Catharina 1934 [handwritten]/ Type Gschw [handwritten]/ Coll. Gschwendtner/ Plaumanni Gsch. [handwritten] det. Gschwendtner/ Type [red label].” This single specimen with the red type label is hereby designated as lectotype for the series (Figs 21–23). The other four specimens become paralectotypes and are mounted on two pins, two are labeled, “Brasilien, St Catharina 1934 [handwritten with black line border]/ Coll Gschwendtner/ Amarodytesplaumanni Gschw Det. Gschwendt. [first three words handwritten]/ Paratype Gschw [brown label with black line border]/ Paratype [handwritten, red label, lower right corner torn away]; other two labeled, “Brasilien, St Catharina 1934 [handwritten label with black line border]/ Amarodytesplaumanni Gschw Det. Gschwendt. [first three words handwritten]/ plaumanni Gschw. det. Gschwendtner [two of these labels]/ Coll. Gschwendnter [three of these labels]/ Paratype Gschw [brown label with black line border]/ Paratype [handwritten, red label].” No additional specimens were examined.

### ﻿ Miradessus pulchellus

Taxon classificationAnimaliaColeopteraDytiscidae

(Guignot, 1955) comb. nov.

D5760362-BB6E-5CDC-9CAF-82BC4D9755C1

Figs 3 , 7 , 13 , 14 , 17 , 24–26 , 30

Amarodytes pulchellus Guignot, 1955: 273; Young 1969: 2; Trémouilles 1995: 47; Nilsson and Hájek 2022: 101.

#### Type locality.

Colombia.

#### Diagnosis.

See above under the very similar *M.beni* for diagnosis.

#### Description.

***Measurements*.**TL = 2.6–2.8 mm, GW = 1.3–1.4 mm, PW = 1.0–1.2 mm, HW = 0.6–0.8 mm, EW = 0.3–0.4 mm, TL/GW = 1.9–2.1, HW/EW = 1.7–1.8, FL/RL = 2.2–2.3.

***Habitus*.** Body shape elongate-oval, lateral outline somewhat discontinuous between pronotum and elytron, posterior apex narrowly rounded (Fig. 3).

***Coloration*** (Fig. 3). Head yellow-orange. Pronotum yellow-orange with a narrow, rounded lobe of black broadly along each side of posterior margin. Elytron maculate, yellow-orange and black, margins of maculae strongly demarcated; with large, transverse yellow-orange regions anteriorly, medially, and apically, not extending medially to suture, with narrow band of black along entire length of suture, margins of maculae irregular and variously lobed. Ventral surfaces mostly dark orange-brown, legs, epipleuron, and ventral areas of prothorax and head lighter orange-yellow, some sutural margins darker, infuscate, mesothoracic ventrites and prosternal process strongly infuscate to nearly black.

***Sculpture and structure*.** Head shiny and smooth, nearly impunctate, with small micropunctures sparsely distributed; eyes large (HW/EW = 1.7–1.8); antennae slender, unmodified. Pronotum with lateral margins moderately curved anteriorly, shallowly curved posteriorly; with narrow bead along entire margin; surface shiny, moderately and evenly punctate; lateral pronotal plica weakly impressed, sublinear, extending about 1/3 distance across pronotum. Elytron with lateral margin evenly and broadly rounded; surface shiny, moderately, and evenly punctate. Prosternum medially moderately broad, medially not protruberant, mediolaterally somewhat granular; prosternal process moderately broad medially with slight tubercle, apical blade large, with fine setae, laterally with low rounded ridges along entire length, medially longitudinally shallowly sulcate, lateral margins slightly convergent to narrowly rounded apex (Fig. 7). Metaventrite and metaventral wings smooth and shiny, covered with shallow punctures. Metacoxa with medial portion moderately broad, metacoxal lines distinct, evenly divergent anteriorly to posterior margin of metaventrite; lateral portion large, medially and anteriorly covered with shallow punctures; metatrochanter about 1/3 length of metafemur (Fig. 7). Abdominal ventrites impunctate except II and III laterally with shallow, indistinct punctures; VI strongly concave in lateral aspect, apex pointed.

***Male genitalia*.** Median lobe in ventral aspect broad, trilobed, with medial, dorsal elongate slender, apically pointed portion and ventral, elongate apically bilobed portion, with each ramus elongate and apically truncate, medial dorsal portion nearly as long as ventral portions (Fig. 13); lateral lobe in lateral aspect robust, basal segment moderately broad, curved, apical segment elongate, slightly curved ventrad, apex rounded (Fig. 14).

***Female genitalia*** (Fig. 17). Bursa copulatrix short; spermathecal duct extremely long, slender, somewhat coiled, broadly expanded in elongate section before receptacle; receptacle similar in size to spermatheca, intermediate duct between receptacle and spermatheca broad, short; spermatheca spherical with broad, somewhat more sclerotized expansion at opening to spermathecal duct, spermathecal spine prominent, elongate, and broad; fertilization duct slender, irregularly curved, heavily sclerotized.

***Sexual dimorphism*.** Males have the pro- and mesotarsomeres I–III slightly but distinctly broader than in females. Abdominal ventrite VI strongly concave in lateral aspect in females, but medially somewhat swollen and apically with a broadly rounded depression in males.

***Variation*.** There is some minor variation in extent of coloration of the dorsal surface, but otherwise specimens are similar.

#### Distribution.

*Miradessuspulchellus* was described from Colombia, without greater specificity. Specimens were examined from numerous localities throughout northwestern Venezuela (states of Barinas, Guarico, Lara, Portuguesa, Tachira, Trujillo, and Zulia) (Fig. 30).

#### Habitat.

Specimens have been collected mainly from exposed and sunny areas in lotic margins (small rivers and streams) and nearby pools. They are often numerous in these habitats.

#### Material examined.

***Type specimens*. *Holotype*** male (Figs 24–26) in ZSMG labeled, “pulchellus in Colomb [HW]/ Sammlung C.L. Müller/ Amarodytes nov. speci. [handwritten]/ Type [red label]/ Holotypus [red label]/ Dr. F. Guignot det., 1955 Amarodytespulchellus n. sp. Type. [handwritten].” The holotype has the male genitalia and apical abdominal segments dissected. No other type specimens accompany the holotype in ZSMG (D.A. Balke pers. comm.).

***Other material examined*.** 134 total examined, all from Venezuela (SEMC), with the following data (SEMC accession numbers in Table 1); 7, Barinas, Rio Caramuca, E of El Corozo, 8°35.449'N, 70°19.427'W, 213 m, 14 Jul 2009, Short et al., river margins, VZ09-0714-04A; 1, Barinas, Rio Paguey at Los Rozos, 8°30.764'N, 70°27.233'W, 190 m, 24 Jan 2012, Short, Arias and Gustafson, river margins, VZ12-0214-03A; 7, Barinas, Rio Sta Barbara, E Sta Barbara, 7°50.028'N, 71°11.188'W, 177 m, 26 Jan 2012, Short, Arias and Gustafson, sandy sidepool in floodplain, VZ12-0126-01B; 6, Barinas, river nr Bum Bum, 8°18.033'N, 70°45.201'W, 216 m, 15 Jul 2009, Short et al., river margins, VZ09-0715-02A; 40, Guarico, Rio San Antonio, N Dos Caminos, 9°46.320'N, 67°21.177'W, 280 m, 8 Jan 2009, Miller and Short, side stream, VZ09-0108-02B; 6, Guarico, Rio San Antonio, N Dos Caminos, 9°46.320'N, 67°21.177'W, 280 m, 8 Jan 2009, Short, Miller, García, Camacho and Joly, river margin, VZ09-0108-02A; 4, Guarico, Rio San Antonio, N Dos Caminos, 9°46.320'N, 67°21.177'W, 280 m, 8 Jan 2009, Short, Miller and García, river margin, VZ09-0108-02A; 39, Lara, Rio Salado, W of Arenales, 10.15433333°N, 69.95763333°W, 490 m, 22 Jan 2009, Short, Camacho, Garcia, gravel stream, VZ09-0122-01X; 1, Portuguesa, Rio Are at Aparición, 9°22.900'N 69°23.153'W, 220 m, 22 Jan 2012, Short, Arias, river margins, VZ12-0122-02A; 3, Portuguesa, Aparición by highway, 9°22.268'N, 69°23.062'W, 213 m, 22 Jan 2012, Short, Arias & Gustafson, roadside pond, VZ12-0122-01A; 10, Trachira, El Tamá National Park, 7°35.038'N, 72°10.340'W, 472 m, 16 Jul 2009, Short, Sites, García, Inciarte, Gustafson and Camacho, HG Vapor light, VZ09-0716-07A; 3, Trujillo, Rio Jiripara nr. Sabana Grande, 9°42.307'N, 70°32.570'W, 199 m, 29 Jan 2012, Short, river margins, VZ12-0129-02B; 6, Trujillo, Rio Jiripara nr Saban Grande, 9°42.307'N, 70°32.570'W, 199 m, 29 Jan 2012, Short, Arias and Gustafson, muddy pool in floodplain, VZ12-0129-02A; 1, Zulia, Perija NP, Tukuko, Rio Tukuko, 9°50.513'N, 72°48.334'W, 252 m, 5 Jul 2009, Short and Gustafson, riffle/rocks in river, VZ09-0705-01B.

**Table 1.** SEMC (University of Kansas) accession numbers for *Miradessuspulchellus* specimens.

Species	Accession numbers
*Miradessuspulchellus*	SEMC1029297, SEMC1029305, SEMC1029315, SEMC1029333, SEMC1029334, SEMC1029343, SEMC1044663, SEMC1044678, SEMC1044683, SEMC1044698, SEMC1044732, SEMC1044735, SEMC1044741, SEMC1044744, SEMC1044745, SEMC1044977, SEMC1044981, SEMC1045044, SEMC1045069, SEMC1045633, SEMC852659, SEMC852661, SEMC852662, SEMC852666, SEMC852669, SEMC852670, SEMC852674, SEMC852675, SEMC852677, SEMC852679, SEMC852680, SEMC852686, SEMC852690, SEMC852695, SEMC852702, SEMC852703, SEMC852708, SEMC852711, SEMC852712, SEMC852723, SEMC852726, SEMC852730, SEMC852745, SEMC852746, SEMC852752, SEMC852753, SEMC852762, SEMC852763, SEMC852765, SEMC852768, SEMC852783, SEMC852784, SEMC852785, SEMC852786, SEMC852789, SEMC852806, SEMC852816, SEMC852821, SEMC856675, SEMC856676, SEMC856678, SEMC856680, SEMC856681, SEMC856682, SEMC856683, SEMC856685, SEMC856686, SEMC856687, SEMC856688, SEMC856689, SEMC856692, SEMC856693, SEMC856694, SEMC856695, SEMC856696, SEMC856697, SEMC856699, SEMC856700, SEMC856701, SEMC856702, SEMC856703, SEMC856704, SEMC856707, SEMC856718, SEMC856720, SEMC856721, SEMC856724, SEMC856726, SEMC856727, SEMC856728, SEMC856734, SEMC856737, SEMC856738, SEMC856739, SEMC856740, SEMC856758, SEMC856759, SEMC856760, SEMC856761, SEMC857535, SEMC857537, SEMC857548, SEMC857550, SEMC857551, SEMC857552, SEMC864033, SEMC864036, SEMC864077, SEMC864082, SEMC875224, SEMC875225, SEMC875247, SEMC875248, SEMC875250, SEMC875254, SEMC876023, SEMC876024, SEMC876056, SEMC876058, SEMC876060, SEMC876064, SEMC876078, SEMC876080, SEMC876095, SEMC876313, SEMC879014, SEMC880761, SEMC880779, SEMC880782, SEMC880785, SEMC880799, SEMC880802, SEMC880810

### ﻿ Miradessus rikae

Taxon classificationAnimaliaColeopteraDytiscidae

Miller, Benetti & Michat sp. nov.

B983A136-6A14-56A0-8AFB-68A7C53FB4A3

https://zoobank.org/DE04E790-A93F-4ED8-8D63-4B6BA7653AE3

Figs 4 , 8 , 15 , 16 , 27–29 , 30

#### Type locality.

Ecuador, Pastaza, Puyo.

#### Diagnosis.

This species is shorter and more robust (Fig. 4) than either *M.pulchellus* or *M.beni* (Figs 1, 3) although the dorsal color pattern is similar to them (Fig. 4). The male genitalia are diagnostic. The median lobe in *M.rikae* is trilobed like other *Miradessus*, but the median portion is uniquely short, broad, and apically broadly subtruncate with the ventral portions elongate, slender and apically narrowly rounded (Fig. 15).

#### Description.

***Measurements*.**TL = 2.2 mm, GW = 1.2 mm, PW = 0.9 mm, HW = 0.6 mm, EW = 0.4 mm, TL/GW = 1.8, HW/EW = 1.5, FL/RL = 2.0.

***Habitus*.** Body shape elongate-oval, lateral outline discontinuous between pronotum and elytron, posterior apex narrowly rounded (Fig. 4).

***Coloration*** (Fig. 4). Head yellow-orange. Pronotum yellow-orange with a narrow, rounded lobe of black broadly along each side of posterior margin. Elytron maculate, yellow-orange and black, margins of maculae strongly demarcated; with large, transverse yellow-orange regions anteriorly, medially and apically, not extending medially to suture, with narrow band of black along entire length of suture, margins of maculae irregular and variously lobed. Ventral surfaces mostly orange; legs, epipleuron, and lateral areas of prothorax and head lighter orange-yellow.

***Sculpture and structure*.** Head shiny and smooth, nearly impunctate, with small micropunctures sparsely distributed; eyes large (HW/EW = 1.5); antennae slender, unmodified. Pronotum with lateral margins moderately curved; with narrow bead along entire margin; surface shiny, moderately and evenly punctate; lateral pronotal plica distinctly impressed, sublinear, extending about 1/3 distance across pronotum. Elytron with lateral margin strongly curved anteriorly in dorsal aspect, evenly curved to apex; surface shiny, moderately and evenly punctate. Prosternum medially moderately broad, medially not protruberant, mediolaterally somewhat granular; prosternal process moderately broad medially with slight tubercle, apical blade large, laterally with prominent rounded ridges along entire length, medially longitudinally sulcate, lateral margins slightly convergent to narrowly rounded apex. Metaventrite and metaventral wings smooth and shiny, covered with shallow punctures (Fig. 8). Metacoxa with medial portion moderately broad, metacoxal lines distinct, evenly divergent anteriorly to posterior margin of metaventrite; lateral portion large, medially and anteriorly covered with small, shallow punctures; metatrochanter about 1/3 length of metafemur (Fig. 8). Abdominal ventrites nearly impunctate except II and III laterally with shallow, indistinct punctures; VI with apex pointed.

***Male genitalia*.** Median lobe in ventral aspect conspicuously trilobed, with medial portion short and broad, apically expanded and subtruncate, apicolateral angles with short setae, ventral portions elongate, slender, apically narrowly rounded (Fig. 15); lateral lobe in lateral aspect robust, basal segment somewhat broad and robust, apical segment broad basally, with deep emargination along dorsal margin, apex rounded with series of setae (Fig. 16).

***Sexual dimorphism*.** Only males are known.

***Variation*.** The two specimens exhibit slight variation in the shape and extent of maculation on the dorsal surface, but they are otherwise similar.

#### Etymology.

This species is named *rikae* after Ms Rikelle Timpe, close friend of the first author.

#### Distribution.

*Miradessusrikae* is known from two sites in Ecuador (Fig. 30).

#### Habitat.

The two known specimens were collected at blacklights, so nothing is known of the specific habitat. The two collection localities are in forested regions of lowland Ecuador.

#### Material examined.

***Type material*. *Holotype*** male (Figs 27–29) in USNM labeled, “ECUADOR,Past. Puyo. 1-7 Feb. 1976 at blacklight Spangler,et al./ ECUADOR-PEACE CORPS- SMITHSONIAN INSTITUTION AQUATIC INSECT SURVEY/ HOLOTYPE *Miradessusrikae* Miller, Benetti & Michat, 2023 [red label with double black line border].” One paratype male in USNM labeled “ECUADOR,NAPO, Lago Agrio(5 Km N) 26 Sept.1975 at blacklight Andrea Langley/ PARATYPE *Miradessusrikae* Miller, Benetti & Michat, 2023 [blue label with black line border].” Both the holotype and paratype have the male genitalia dissected and placed in microvials mounted on the pins.

### ﻿Key to species of *Miradessus*

**Table:** 

1	Anterior surface of head brown (Fig. 2); surface of metacoxa densely covered with punctation (Fig. 6); ventral portion of male median lobe approximately unilobate (Fig. 11)	***Miradessusplaumanni***
–	Anterior surface of head yellow-orange (Figs 1, 3, 4); surface of metacoxa with punctation sparsely distributed (Fig. 5, 7, 8); ventral portion of male median lobe apically strongly bilobate, each ramus elongate and relatively slender (Figs 9, 13, 15)	**2**
2	Body short and robust (Fig. 4; TL/GW = 1.8); male median lobe with medial portion broad and apically subtruncate (Fig. 15)	***Miradessusrikae***
–	Body more elongate and slender (Figs 1, 3; TL/GW = 1.9–2.1); male median lobe with medial portion slender and apically narrowly rounded (Figs 9, 13)	**3**
3	Male median lobe with medial portion elongate, nearly as long as ventral portions, with distinct laterally divergent pencils of setae apically (Fig. 13); lateral lobes with apical segment nearly as long as basal segment, curved ventrad (Fig. 14)	***Miradessuspulchellus***
–	Male median lobe with medial portion much shorter than ventral portions, apex with series of setae (Fig. 9); lateral lobes with apical segment only about 1/3 length of basal segment, curved dorsad (Fig. 10)	***Miradessusbeni***

### ﻿Species in the genus *Miradessus*

*Miradessusbeni* Miller, Benetti & Michat, sp. nov. (Bolivia, Peru, Fig. 30)

*Miradessusplaumanni* (Gschwendtner, 1935), comb. nov. (Brazil, Fig. 30)

*Miradessuspulchellus* (Guignot, 1955), comb. nov. (Colombia, Venezuela, Fig. 30)

*Miradessusrikae* Miller, Benetti & Michat, sp. nov. (Ecuador, Fig. 30)

## Supplementary Material

XML Treatment for Miradessus

XML Treatment for Miradessus beni

XML Treatment for Miradessus plaumanni

XML Treatment for Miradessus pulchellus

XML Treatment for Miradessus rikae

## ﻿Additional information

### Author ORCIDs

Cesar J. Benetti https://orcid.org/0000-0001-6795-2194

Mariano C. Michat https://orcid.org/0000-0002-1962-7976

### Data availability

All of the data that support the findings of this study are available in the main text.
